# Prevalence of metabolic syndrome in schizophrenia patients treated with antipsychotic medications

**DOI:** 10.22088/cjim.11.3.310

**Published:** 2020-05

**Authors:** Nayyer Jaberi, Elnaz Faramarzi, Mostafa Farahbakhsh, Alireza Ostadarahimi, Mohammad Asghari Jafarabadi, Ali Fakhari

**Affiliations:** 1 Research Center of Psychiatry and Behavioral Sciences, Tabriz University of Medical sciences, Tabriz, Iran; 2 Liver and Gastrointestinal Diseases Research center, Tabriz University of Medical sciences, Tabriz, Iran; 3 Nutrition Research Center, Tabriz University of medical sciences, Tabriz, Iran; 4 Medical Education Research Center, Department of Statistics and Epidemiology, Faculty of Health, Tabriz University of Medical Sciences, Tabriz, Iran

**Keywords:** Schizophrenia, Metabolic syndrome, Abdominal obesity, Hypertention

## Abstract

**Background:**

This study was conducted to determine the prevalence of metabolic syndrome (Mets) in schizophrenic patients in a 6-month period of treatment with antipsychotic medications.

**Methods:**

In this study, 60 volunteer schizophrenic patients were included. At the onset and 6 months after treatment with antipsychotic medications, fasting blood sugar (FBS), serum triglyceride (TG), high density lipoprotein (HDL), weight, waist circumference (WC), and blood pressure were determined. We defined Mets according to ATPIII criteria.

**Results:**

After a 6-month treatment with antipsychotic drugs, the mean WC, serum TG, HDL, systolic and diastolic blood pressure increased but the changes of WC and HDL were statistically significant (p<0.05). We found that the percentage of patients with high WC, low HDL levels, and Mets increased after treatment which was statistically significant (p<0.05).

**Conclusion:**

It is recommended that nutritional and lifestyle changes intervention should be implanted for schizophrenic patients undergoing treatment.

Schizophrenia is a neurodevelopmental disorder that affects 1% of the population around the world. Patients with schizophrenia have a lower life expectancy than the general population (1, 2). Epidemiological studies have found a relationship between schizophrenia and increased prevalence of type 2 diabetes and cardiovascular diseases (1). The most commonly reported cause of death in schizophrenia is cardiovascular diseases (2). Increased mortality from cardiovascular causes in schizophrenic patients is associated with various factors such as genetic vulnerability, exposure to cigarette smoke, sedentary lifestyle, antipsychotic medications, etc. A major risk factor for cardiovascular diseases in schizophrenia is metabolic syndrome (MetS) that is highly prevalent in schizophrenic patients (3). Unfortunately, the symptoms of this syndrome are often not diagnosed. The causes of MetS are not well known in schizophrenic patients (4), and recent researches have shown that the incidence of MetS in schizophrenic patients has increased (1). The incidence of MetS differs by country and in psychiatric populations, and its progression is rapid (5). Considering that the results of studies on the incidence of MetS in these patients are contradictory and despite the increasing incidence of MetS and the absence of a report on the prevalence of this syndrome in schizophrenic patients, the present study was conducted to determine the prevalence of MetS in schizophrenic patients over a 6-month period of treatment with antipsychotic medications. 

## Methods

This descriptive study was approved by the Ethics Committee of the Tabriz University of Medical Sciences (ID number 008819). 

In this study, 60 volunteer schizophrenic patients who were referred to the Bozorghmehr Outpatient Clinic were included. After explaining the study protocol for the patients, they signed a written informed consent. Inclusion criteria included patients with schizophrenia (with a semi-structured clinical interview based on DSM-IV and a psychiatric diagnosis). Exclusion criteria included patients who had been previously treated with psychiatric drugs for more than a month, patients treated with lipid-lowering drugs and hypertension, patients with liver, kidney, diabetes, or thyroid diseases, cancer, or other hormonal disorders. Demographic information, including age, gender, marital status, and educational level was collected via a questionnaire.

**Biochemical factors: **Blood samples were taken after an overnight fast of 12 hours. Fasting blood sugar (FBS), serum triglyceride (TG), and high-density lipoprotein (HDL) were assessed using Pars Azmoon kits by the enzymatic method.

**Anthropometric measurements: **Height and weight were measured by mounted tape (the nearest to 1 mm) and Seca scale (the nearest 0.1 kg) respectively. Body mass index (BMI) was calculated by dividing weight (kg) by the square of height (m).

According to National Institutes of Health (NIH) guidelines, waist circumference (WC) was measured. Women with a waist circumference ≥88 cm and men with a waist circumference of ≥102 cm were considered abdominally obese. 

**Blood pressure measurements: **In the sitting position, blood pressure was measured twice in each arm. There was a two minute rest between each measurement. The average of the two measurements in each arm was reported as blood pressure. 

**Metabolic syndrome definition: **MetS is defined according to the National Cholesterol Education Program’s Adult Treatment Panel III report (ATPIII) criteria (6). 

Subjects with three or more of the following conditions were defined as having MetS: WC ≥102 cm in men and ≥88 cm in women, TG≥150 mg/dl (drug treatment for elevated triglycerides was used as an alternate indicator), HDL-C <40 mg/dl in men and <50 mg/dl in women; elevated blood pressure (systolic ≥130 and/or diastolic ≥85 mm Hg; antihypertensive drug treatment in a patient with a history of hypertension was used as an alternate indicator); and elevated fasting glucose≥100 (drug treatment of elevated glucose was used as an alternate indicator).

**Statistical analysis: **Data were analyzed by SPSS Version 11.5 (Chicago, IL). Descriptive statistics were performed for all study variables and are presented as mean±SD as well as number (percentage) where applicable. To compare variables before and after treatment, paired-t-tests and McNemar’s test were used to for quantitative and qualitative variables, respectively. Statistical significance was considered to be p<0.05.

## Results

The baseline and demographic characteristics of the patients are indicated in table 1. Of the 60 patients, 12 (20%) patients were females and 48 (80%) of patients were males. Fifteen (25%) patients smoked cigarettes and two (3%) patients used opium. The mean age of patients was 41.25±7.58 years old. As shown in table 2, after a 6-month period of treatment with antipsychotic medications, mean WC, serum TG, HDL, systolic and diastolic blood pressure increased. The changes in WC and HDL were statistically significant (p<0.05). The percentage of patients with high WC (28.3% vs 48.3%), low HDL levels (65% vs 93.3%), and MetS (5% vs 26.7%) increased following the treatment, and all changes were statistically significant (p<0.05). As indicated in table 4, there is no statistical difference in the type of drugs used in patients with metabolic syndrome and without it (P=0.9). 

**Table1:** Baseline characteristics of patients

**Variable **	**N (%)**
Gender	
Female	12 (20(
Male	48 (80)
Marital status	
Single	14(23.3)
Married	44(73.3)
Education level	
<diploma	28(46.7)
Diploma	25(41.7)
College	7(11.7)
Cigarette smoking	15(25)
Opium use	2(3.3)
Alcohol	0
First generation antipsychotic medication	13(21.7)
Second generation antipsychotic medication	34(56.7)
First generation antipsychotic medication+mood stabiliser	5(8.3)
Second generation antipsychotic medication+ mood stabiliser	8(13.3)

**Table2:** Comparison of the mean of metabolic syndrome components before and after treatment (n=60)

	**Before treatment**	**After treatment**	*P
	**mean±SD**	**mean±SD**	
Waist circumference (cm)	94.73±8.58	96.91±7.88	0.009
Fasting blood sugar(mg/dl)	88.36±15.43	88.93±12.44	0.79
Triglyceride (mg/dl)	140.15±74.22	152.36±89.53	0.09
High density lipoprotein (mg/dl)	39.38±12.52	35.03±6.99	0.007
Systolic blood pressure (mmHg)	112.75±6.91	114.75±9.40	0.15
Diastolic blood pressure (mmHg)	70±8.18	72.75±8.55	0.08

*P: paired t test

**Table 3:** Comparison of the percentage of patients with metabolic syndrome components and metabolic syndrome before and after treatment (n=60)

**Variables **	**Before treatment**	**After treatment**	*P
	**N(%)**	**N(%)**	
Hypertension	2(3.3)	4(6.7)	0.68
Fasting blood sugar (≥100mg/dl)	4(6.7)	6(10)	0.68
Triglyceride (≥150mg/dl)	21(35)	26(43.3)	0.26
High density lipoprotein (male <40 ;female <50 mg/dl)	39(65)	56(93.3)	<0.001
Waist circumference (male ≥102;female≥88 cm)	17(25)	29(48.3)	0.0005
Metabolic syndrome	3(5)	16(26.7)	0.001

*P: McNemar test

**Table 4:** Comparison of the percentage of patients treated with different antipsychotic drugs

	**Metabolic syndrome**	
	**Yes**	**No**
	**N(%)**	**N(%)**
first generation antipsychotic medication	3(18.8)	10(27.7)
Second generation antipsychotic medication	9(56.3)	25(56.8)
first generation antipsychotic medication+mood stabilizer	2(12.5)	3(6.8)
Second generation antipsychotic medication+ mood stabilizer	2(12.5)	6(13.6)

*Chisquare

## Discussion

 In this descriptive study, after a 6-month period of treatment of schizophrenia patients with antipsychotic medications, the prevalence of MetS and increases in serum HDL and WC as MetS components were statistically significant. Moreover, the percentage of patients with MetS at the beginning of the study was 5% that had increased to 27% after 6 months of treatment .These results are supported by another study which found that about 25% of Iranian patients with schizophrenia had MetS (7). Results of studies from other countries reflect similar incidences of metabolic syndrome. For example, the prevalence of MetS in patients with schizophrenia in Turkey was 34% (8), in Spain 39% (9), in Singapore 46% (10), in Egypt 38%, (11) and in Japan about 28%(12). Differences in these findings among countries may be due to differences in the socio-demographic profiles, definitions of metabolic syndrome and patients’ lifestyles. The precise causes of the high prevalence of MetS in schizophrenia patients are not well-known. Some mechanisms have been proposed. First, in these patients, poor dietary habits as well as a sedentary lifestyle due in part to side effects of antipsychotic medications and the effect of antipsychotic medications on metabolism increases the risk of obesity, especially abdominal obesity (13-15). Our findings indicated waist circumference increased significantly after 6 months of treatment, which supports this hypothesis. Secondly, hypercortisolemia due to abnormalities of the hypothalamic-pituitaryadrenal (HPA) axis (16) might also be involved in higher risk of MetS in these patients. In addition, we found that the percentage of patients with hypertension did not alter significantly after treatment. Because of the sedative effect of antipsychotics, (e.g., risperidone's anti-alpha1 adrenergic effect), blood pressure may not change during the course of treatment with antipsychotic medications. 

In contrast to other studies, which reported that the incidence of diabetes was higher in schizophrenia patients in comparison with the general population (17, 18), our results showed that the percentage of patients with FBS ≥100 mg/dl following treatment did not change compared to the beginning of the study. These differences may be due to the short duration of our study (6 months) as compared with other studies (over 1 year). In this study, the percentage of patients with low HDL increased significantly, which is similar to previous studies (2, 3). A limitation of this study is the small sample size. One strength of this study is that changes in MetS components in schizophrenia patients after 6 months of antipsychotic treatment was compared with the same patients pre-treatment. In most previous studies, the incidence of MetS in schizophrenic patients is compared with the incidence in healthy subjects. 

In conclusion, the percentage of schizophrenia patients with MetS, high WC, and low HDL increased significantly after a 6-month antipsychotic medication regimen. Therefore, patients with schizophrenia, when undergoing polypharmaceutical treatment, should be regularly monitored for metabolic syndrome risk factors, and nutritional and lifestyle interventions should be implemented for schizophrenic patients undergoing treatment with antipsychotic medications. 
